# Study on Typhoon Characteristic Based on Bridge Health Monitoring System

**DOI:** 10.1155/2014/204675

**Published:** 2014-06-10

**Authors:** Xu Wang, Bin Chen, Dezhang Sun, Yinqiang Wu

**Affiliations:** ^1^State Key Laboratory Breeding Base of Mountain Bridge and Tunnel Engineering, Chongqing Jiaotong University, Chongqing 400074, China; ^2^College of Civil Engineering and Architecture, Zhejiang University, Hangzhou 310058, China; ^3^Institute of Engineering Mechanics, China Earthquake Administration, Harbin 150080, China; ^4^Bureau of Public Works of Shenzhen Municipality, Shenzhen 518006, China

## Abstract

Through the wind velocity and direction monitoring system installed on Jiubao Bridge of Qiantang River, Hangzhou city, Zhejiang province, China, a full range of wind velocity and direction data was collected during typhoon HAIKUI in 2012. Based on these data, it was found that, at higher observed elevation, turbulence intensity is lower, and the variation tendency of longitudinal and lateral turbulence intensities with mean wind speeds is basically the same. Gust factor goes higher with increasing mean wind speed, and the change rate obviously decreases as wind speed goes down and an inconspicuous increase occurs when wind speed is high. The change of peak factor is inconspicuous with increasing time and mean wind speed. The probability density function (PDF) of fluctuating wind speed follows Gaussian distribution. Turbulence integral scale increases with mean wind speed, and its PDF does not follow Gaussian distribution. The power spectrum of observation fluctuating velocity is in accordance with Von Karman spectrum.

## 1. Introduction

Typhoon disaster is a major disaster in China, which has caused serious property and casualty losses annually and threatened sustainable development along the east coast of China. To have a better understanding of bridge destruction reasons from typhoon, wind characteristics near ground during typhoon attack must be studied firstly. However, experimental simulation of typhoon is very difficult because of its particularity. This has led to field measurement that has been recognized as the most effective research method and long-term direction in wind-resistant research of structure [1]. A huge mass of data was accumulated and parts of research result had been used in model of conduct in developed countries, where the field-measured research in strong wind characteristics developed earlier. Database for wind characteristics had been built in some countries, where the research of wind engineering is developed [2–6]. China's field-measured research of wind characteristics developed later but very fast; some valuable research results had been achieved in recent years [7–12]. Currently, most researches on near ground wind characteristics during typhoon attack focus on buildings; few have been performed on bridges, but it is important for wind engineering research.

In China, health monitoring systems have been installed on many long span bridges [13–16] to capture health states of bridges. Among all these systems, the health monitoring of wind load is of primary importance to bridge health monitoring systems because the health monitoring information gathered could be used in bridge design and construction. However, current practices of strong wind characteristics using wind load monitoring system on bridge site are still encountering shortage.

Based on the wind data measured from the anemometer positioned on Jiubao Bridge at 6 m height, Hangzhou city, near-ground wind characteristics under typhoon HAIKUI in 2012 were discussed in this paper, which include wind speed and direction, gust factor, turbulence intensity, peak factor, turbulence integral scale, and power spectrum of wind speed. Obviously, such studies are useful for promotions of wind-resistant design of bridge in the future.

## 2. Site Description and Measurement Characterization

### 2.1. Introduction of Typhoon HAIKUI

**Figure 1:**
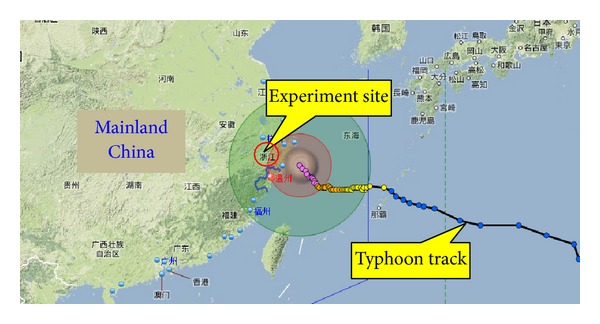
Experiment site and track of typhoon HAIKUI.

In 2012, the 11th tropical storm “HAIKUI” (named by HAIKUI, number 1211 tropical cyclone), named from a Chinese marine animal, headed to the coast of East China Sea at 17:00 on August 17, 2012, and landed at Zhejiang province in the morning of August 8. The location of the eye was 640 km from southwest of Zhejiang province, which was very close to the test bridge. The maximum 10 min mean wind speed at 10 m height was found to be 28 m/s, and the lowest pressure was 980 hPa.  Figure 1 shows a satellite view of the path of typhoon HAIKUI and the experiment site. According to the statistical results of meteorological data from Chinese Ministry of Civil Affairs and Disaster Reduction Office, typhoon HAIKUI caused 6 casualties in Zhejiang, Shanghai, Jiangsu, and Anhui provinces and forced 2.173 million people to evacuate. The most damaged area was Zhejiang province, resulting in 7.001 million people involved, 1.546 million people were forced to move in emergency, and almost 5100 houses were destroyed.

**Figure 2:**
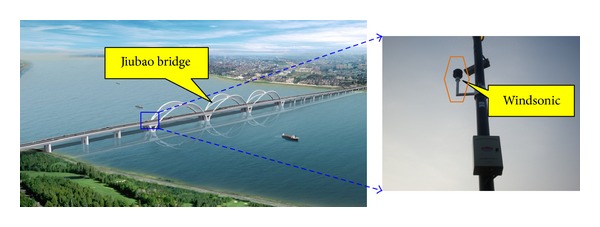
The location and photo of anemometers.

Jiubao Bridge was located on Qiantang River, Hangzhou. The construction work began on December 18, 2009, and was completed in 2012. The bridge was designed to a standard bidirectional and six traffic lanes freeway, combining with 80 km/h of speed limit and about 1855 meters in span length. The whole bridge health monitoring systems which were comprised of vehicle system, wind speed and direction measurement system, and fatigue detection system were installed and distributed on the bridge. The meteorological data of typhoon was detected by a two-dimensional sonic anemometer in the wind speed and direction measurement system (see Figure 2). The anemometer, produced by British Gill Company, was Windsonic and has a sampling frequency of 4 Hz. Wind direction was defined north as 0° along a clockwise direction varying from 0° to 360°. Specifications and parameters of anemometer were considered with the records indicated in Table 1.

## 3. Study of the Field-Measured Wind Data

### 3.1. Mean Wind Speeds and Wind Directions

**Figure 3:**
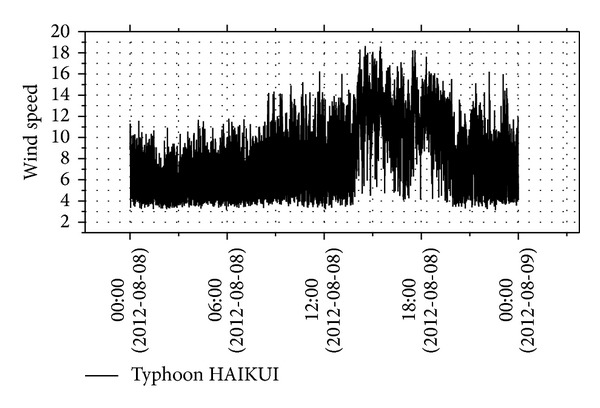
Instantaneous wind speed during typhoon HAIKUI.

**Figure 4:**
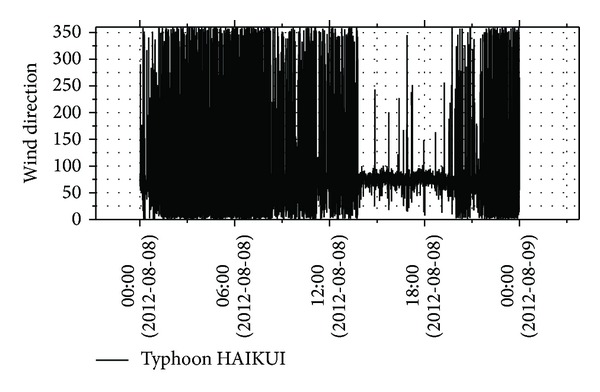
Instantaneous wind direction during typhoon HAIKUI.

Figures 3 and 4 show the time history of wind speeds and directions of typhoon HAIKUI from 0:00 on August 8, 2012, to 0:00 August 9, 2012. Because typhoon HAIKUI did not directly impact the experimental site in lateral direction, an uphill and a downhill of wind speed were observed in the time history, indicating that a peak value was recorded.

**Figure 5:**
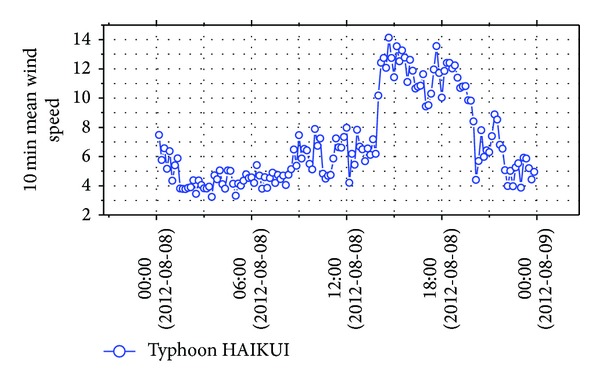
10 min mean wind speed during typhoon HAIKUI.

**Figure 6:**
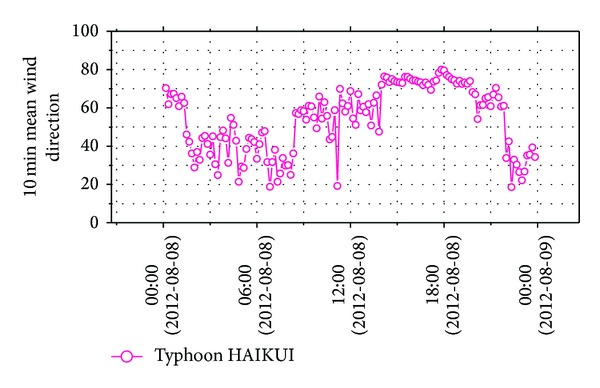
10 min mean wind direction during typhoon HAIKUI.

**Figure 7:**
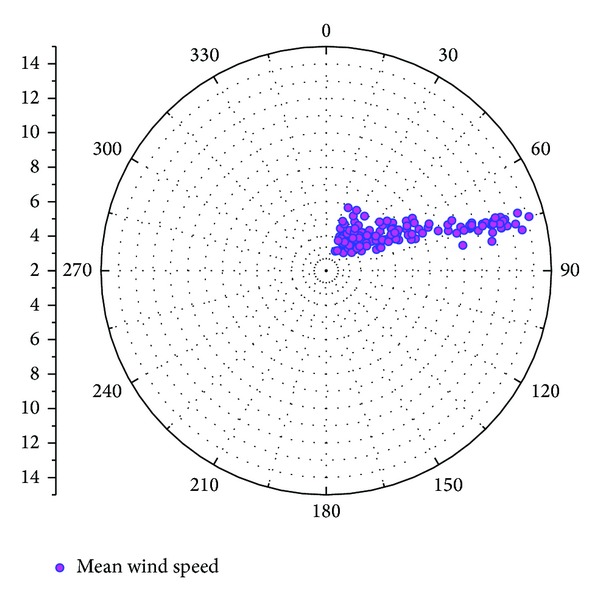
10 min mean wind speed versus mean wind direction.

Before analyzing wind characteristics, the overall sample needs to be separated into several segments by a specified time interval. They are different from regulations of time interval of mean wind speed among standards in many countries, such as 3 s used in America [17] and India [18] and 10 min used in Japan [19], Europe [20], and China [21]. In this paper, according to Chinese standard, wind data was separated into 144 samples, and the maximum 10 min mean wind speed was 14.12 m/s. Figures 5 and 6 show time histories of 10 min mean wind speed and horizontal wind direction. Variations of 10 min mean wind speed with horizontal wind direction were shown in Figure 7, which made the variation trend of mean wind speed with horizontal wind direction intuitive.

After breaking down to 10 min time interval, the wind speed vector was resolved into three components [22], two of which are orthogonal, namely, *u*_*x*_(*t*) and *u*_*y*_(*t*), and can be measured synchronously by a two-dimensional sonic anemometer, denoting directions on *x*-axis and *y*-axis, respectively. The horizontal wind speed *U* and main wind direction *ϕ* are calculated by the following formulas:

(1) $$\begin{array}{c} U=\sqrt{{\bar{u_{x}(t)}}^{2}+{\bar{u_{y}(t)}}^{2},}\\ \cos\left(\phi \right)=\frac{\bar{u_{x}\left(t\right)}}{U},\;\;\sin\left(\phi \right)=\frac{\bar{u_{y}\left(t\right)}}{U},\\ \phi =\text{arccos}\frac{\bar{u_{x}(t)}}{U}+\text{step}\left(-\bar{u_{y}\left(t\right)}\right)\cdot 180^{{}^{\circ}}, \end{array}$$

where step(·) is step function, $\bar{u_{x}(t)}$ is the mean wind speed in 10 min time interval along *X*-direction, and $\bar{u_{y}(t)}$ has the same meaning along *Y*-direction.

The longitudinal fluctuating wind speed *u*(*t*) and lateral fluctuating wind speed *v*(*t*) are obtained from

(2) u(t)=ux(t)cos⁡ϕ(t)+uy(t)sinϕ(t)−U,v(t)=−ux(t)sinϕ(t)+uy(t)cos⁡ϕ(t).

### 3.2. Turbulence Intensity

Turbulence intensity is considered the relative index for intensity of turbulence and then determined as

(3) $$\begin{array}{c} I_{i}=\frac{\sigma_{i}}{U}\;\left(i=u,v,w\right), \end{array}$$

where *σ*_*i*_ is the standard deviation of fluctuating wind speed for component *i*  (*i* = *u*, *v*, *w*).

**Figure 8:**
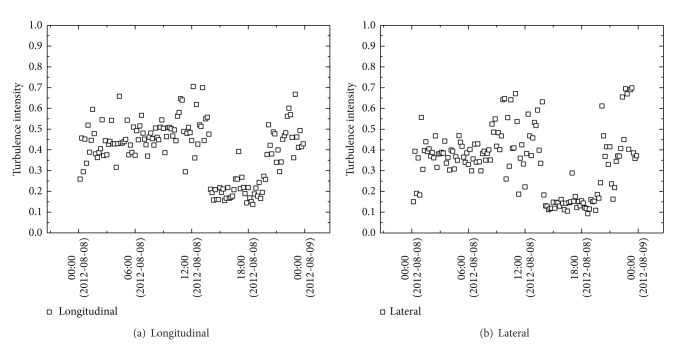
Variation of turbulence intensity during typhoon HAIKUI.

Similar diurnal patterns of 10 min fluctuating wind speed distribution are shown in longitudinal (Figure 8(a)) and lateral (Figure 8(b)) directions. The changes in turbulence intensities are slow between 00:00 and 12:00 on August 8, with average turbulence intensities being 0.47 and 0.40 and maximum values being 0.71 and 0.67 in longitudinal and lateral directions, respectively. In both directions, a sharp decrease is found in the period between 12:00 and 20:00 (the lowest average intensities being 0.16 and 0.13, resp.) before an increase in the period between 20:00 and 00:00 on August 9 (variation ranging from 0.1 to 0.7).

**Figure 9:**
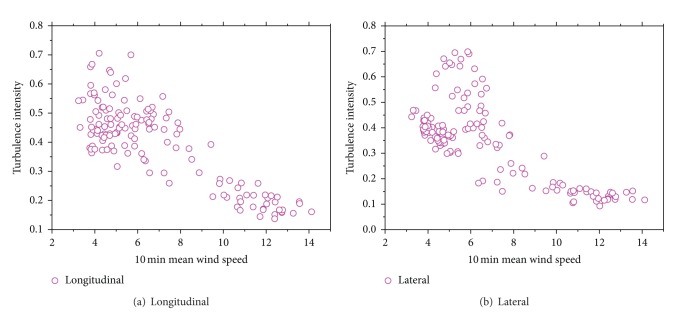
Variation of turbulence intensities with wind speed.

Figure 9 shows both longitudinal and lateral turbulence intensity as a function of 10 min mean wind speed. The overall turbulence intensities reduce with decreased mean wind speed. It is clear from Figure 9 that an obvious decrease is found in turbulence intensities before mean wind speed approaching 8 m/s, but the changes become small when mean wind speed is greater than 8 m/s.

Table 2 shows the ratio of average longitudinal turbulence intensity to average lateral turbulence intensity in both domestic and international field research results. It can be seen from Table 2 that the ratio of the case is slightly larger than that in Peng et al. [12] and Fu et al.'s [23] results but quite close to the results from normal storm wind and typhoon by Li et al. [9], Cao et al. [6], and Shiau and Chen [24–26].

### 3.3. Gust Factor

Gust factor is defined as the ratio of maximum gust wind speed over average gust wind speed. It can be expressed as

(4) Gu(t)=1+max⁡⁡(u(t)¯)U,Gv(t)=max⁡⁡(v(t)¯)U,

where $\max(\bar{u(t)})$ and $\max(\bar{v(t)})$ are the maximum gust wind speed in the period of *t* for longitudinal and lateral fluctuates, respectively.

**Figure 10:**
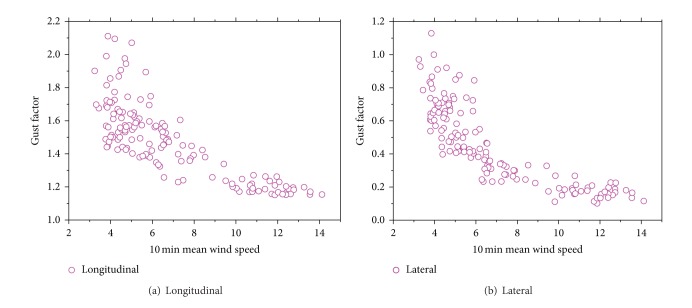
Variation of gust factors with wind speed.

Figure 10 shows the distribution of 3 s gust factor as a function of 10 min mean wind speed both in longitudinal (Figure 10(a)) and lateral (Figure 10(b)) directions. An obvious decrease of gust factor under low wind speed can be seen, but the rate of reduction goes smaller when wind speed becomes high. The average ratio of longitudinal gust factor to lateral gust factor is 0.30.

### 3.4. Peak Factor

There is a similarity in the definition of peak factor as of gust factor. The following expression for peak factor which describes the intensity of fluctuating wind speed is employed:

(5) $$\begin{array}{c} g_{u}=\frac{{\hat{U}}_{t}-U}{\sigma_{u}}, \end{array}$$

where ${\hat{U}}_{t}$ is the maximum value of *t* min average wind speed of the longitudinal component of fluctuating wind velocity record and *σ*_*u*_ is the corresponding standard deviation.

**Figure 11:**
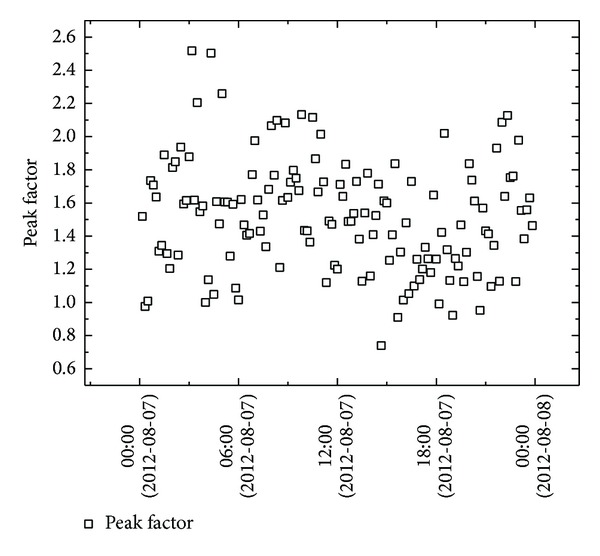
Variation of peak factor during typhoon HAIKUI.

**Figure 12:**
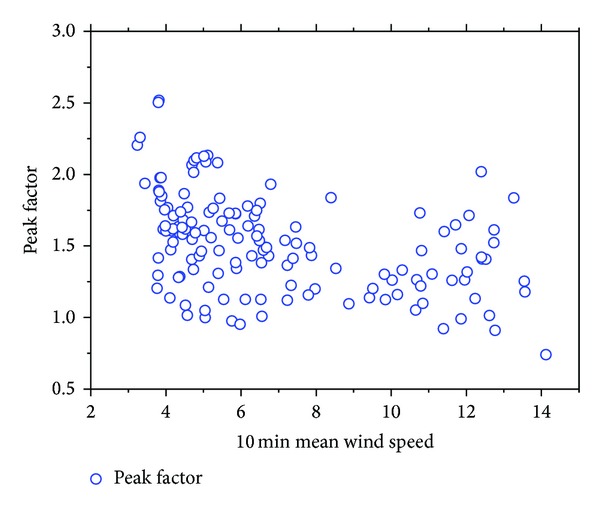
Variation of peak factor with wind speed.

The quantity of peak factor was determined from the three-second averages, shown as a function of time during a one-day period coincident with the passage of typhoon HAIKUI (Figure 11) and 10 min mean wind speed (Figure 12). In each figure, the distribution of the data is in the range of 0.6 and 2.5; the average and standard deviations are 1.52 and 0.34, respectively. A noticeable decrease was found from the analysis of the average peak factor compared with Huang's results [12].

### 3.5. Probability Density Distribution

**Figure 13:**
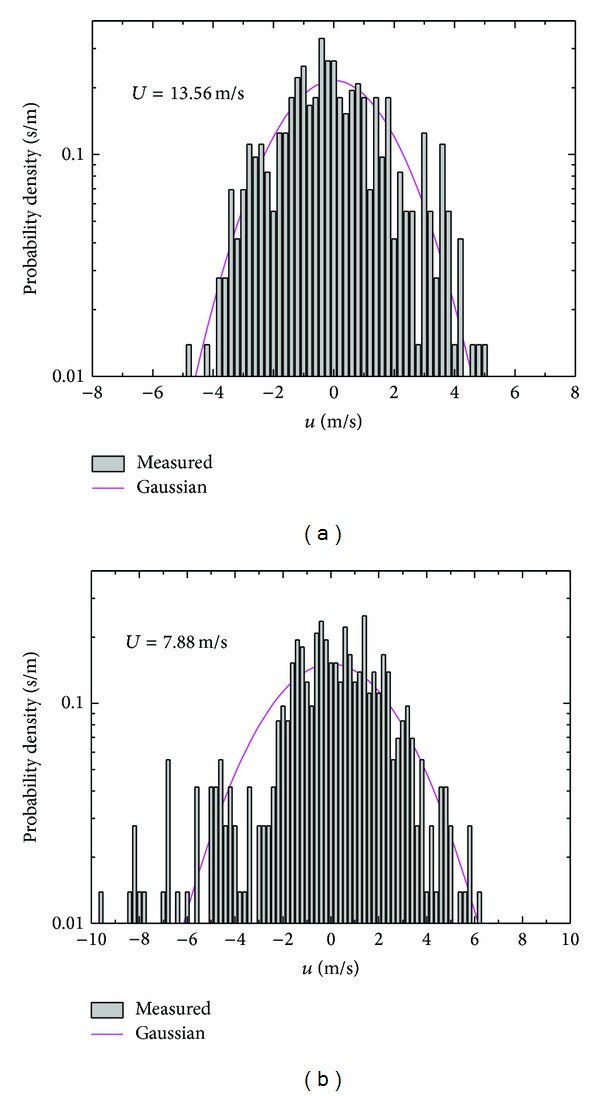
Probability density function of longitudinal wind speed fluctuations.

**Figure 14:**
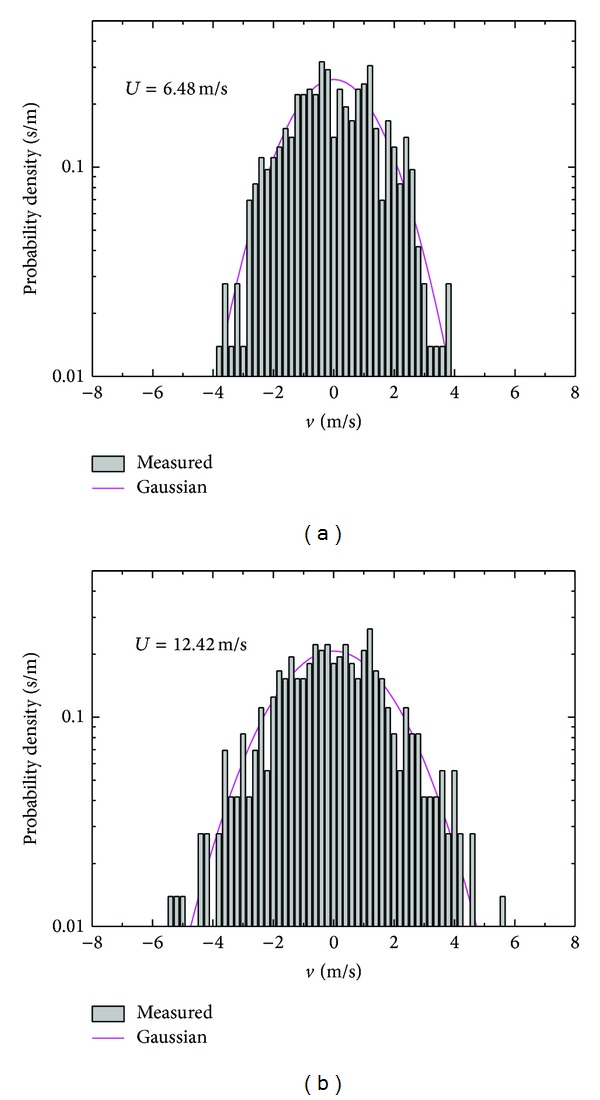
Probability density function of lateral wind speed fluctuations.

The probability density function of wind speed fluctuations is customarily with Gauss hypothesis. However, a similar statistical description of the wind speed fluctuations is generally the lack of certainty when applied to strong typhoon. Analysis of the wind speed fluctuations in longitudinal (Figure 13) and lateral (Figure 14) directions confirms a fairly good agreement with a Gaussian distribution through moment estimation method. It is believed the wind speed fluctuations are examined as Gaussian processes in this region of wind speed.

### 3.6. Turbulence Integral Scale

Turbulence is a three-dimensional spatial structure, and its nine parameters correspond to fluctuating wind speeds in longitudinal *u*, lateral *v*, and vertical *w* directional components. For example, *L*_*u*_^*x*^, *L*_*u*_^*y*^, and *L*_*u*_^*z*^ are the average integral scales of longitudinal-dependent turbulence component fluctuations in longitudinal, lateral, and vertical directions, respectively. Turbulence integral scale *L*_*i*_^*x*^ is mathematically defined as

(6) $$\begin{array}{c} L_{i}^{x}=\frac{1}{\sigma_{i}^{2}}\overset{\infty }{\underset{0}{\int }}R_{i_{1}i_{2}}\left(x\right)dx,\;i=u,v,w, \end{array}$$

where *R*_*i*_1_*i*_2__(*x*) is the covariance function of fluctuating components in two positions.

In general, spatial correlation needs to be transferred into time correlation by Taylor hypothesis due to the fact that simultaneously measuring recorded points in space are complex and difficult. Thus, turbulence integral scale can be calculated by autocorrelation function integral method depending on Taylor hypothesis and interpreted as

(7) $$\begin{array}{c} L_{i}^{x}=\frac{U}{\sigma_{i}^{2}}\overset{\alpha }{\underset{0}{\int }}R\left(\tau \right)d\tau ,\;i=u,v,w, \end{array}$$

where *R*(*τ*) is the autocorrelation function and *α* is the variable when autocorrelation coefficient drops to 0.05 [4].

**Figure 15:**
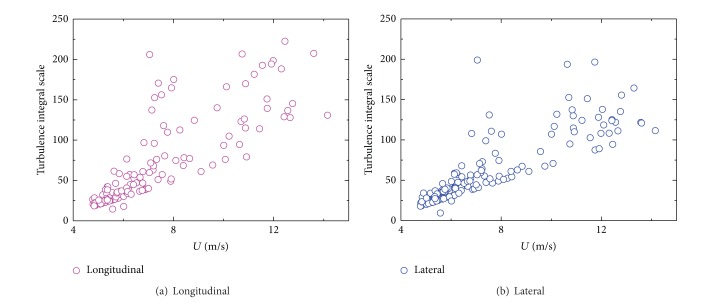
Variation of turbulence integral scale with 10 min mean wind speed.

**Figure 16:**
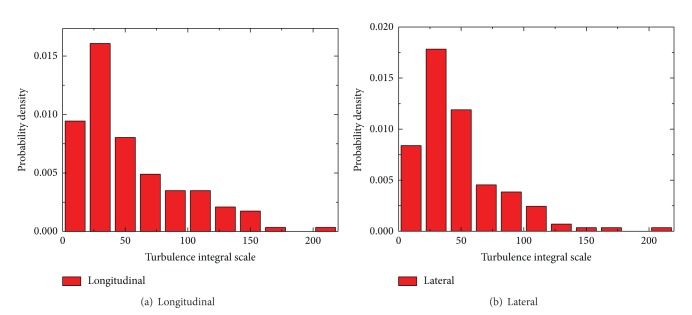
Probability density function of turbulence integral scale.

In Figure 15, turbulence integral scales of longitudinal and lateral components determined from 10 min average show a clear dependency on 10 min mean wind speed, and increasing quantity is recorded by increased 10 min mean wind speed. For 10 min mean wind speed is greater than 8 m/s, turbulence integral scales drastically change and distributed in a large region between 100 and 250. From the statistical analysis of Figure 16 for the turbulence integral scales both in longitudinal (Figure 16(a)) and lateral (Figure 16(b)) directions, a similarity of probability density distribution is observed in each component; as it is obviously asymmetrical and differs from a standardized Gaussian distribution, in particular at low turbulence integral scales which is between 20 and 40, the probability is the largest with most data locate in this region.

### 3.7. Correlation

Autocorrelation describes the correlation of two dependent values of a time series at different moments. *X*(*t*) is here defined as a time series, and the correlation function can be derived as

(8) $$\begin{array}{c} R_{XX}\left(t_{1},t_{2}\right)=E\left[X\left(t_{1}\right)X\left(t_{2}\right)\right]. \end{array}$$

Whereas *X*(*t*) denotes a stationary random process, the correlation function can be derived as

(9) $$\begin{array}{c} R_{XX}\left(\tau \right)=E\left[X\left(t\right)X\left(t+\tau \right)\right], \end{array}$$

where *R*_*XX*_ is the autocorrelation function and *τ* is the delaying time.

Indicating correlation intensity of wind speed fluctuations for different directions, cross-correlation coefficient can be expressed as

(10) $$\begin{array}{c} C_{R(ij)}=\frac{R_{ij}\left(0\right)}{\sqrt{R_{ii}\left(0\right)}\sqrt{R_{jj}\left(0\right)}}, \end{array}$$

where *C*_*R*(*ij*)_ is cross-correlation coefficient of wind speed fluctuations in *i* and *j* directions, *R*_*ij*_ is cross-correlation function of wind speed fluctuations in *i* and *j* directions, and *R*_*ii*_ and *R*_*jj*_ are autocorrelation functions of wind speed fluctuations in *i* and *j* directions, respectively.

**Figure 17:**
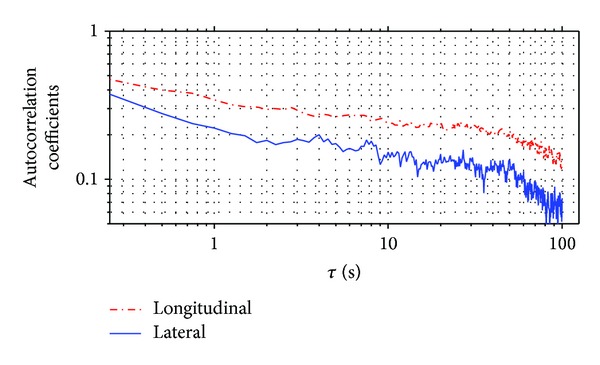
Autocorrelation coefficients of longitudinal and lateral wind speed fluctuation component.

The autocorrelation coefficients for both longitudinal and lateral wind speed fluctuations are plotted in Figure 17 at the average level of 10 min time interval. It is found that a noticeable similarity of tendency is observed from the two plots; in particular with *τ* increases, the autocorrelation coefficients for both directions decrease, whereas the longitudinal autocorrelation coefficient is shown slightly larger than the lateral autocorrelation coefficient at the same delaying time *τ*.

### 3.8. Power Spectra of Wind Speed Fluctuations

Turbulent power spectrum is a describer of turbulent energy distribution in frequency domain and characteristics of wind fluctuation. It can be expressed as [28]

(11) $$\begin{array}{c} \frac{S_{u}\left(n,z\right)}{U_{0}^{*2}}=\frac{A}{{\left(1+Bf^{\beta }\right)}^{\gamma }}, \end{array}$$

where *n* is frequency; *z* is the observed height; *f* denotes reduced frequency derived as *f* = *nz*/*U*(*z*); and *A*, *B*, *β*, and *γ* are undetermined parameters.

Functions of power spectra densities of wind speed fluctuations were expressed in accordance with the Kolmogorov principle as Davenport spectrum, Von Karman spectrum, Simiu spectrum, Kaimal spectrum, and Harris spectrum. Based on the comparisons with field measurement and wind tunnel test, Von Karman spectrum is believed as the best fitted function for wind speed fluctuations. The longitudinal fluctuating component can be expressed as [25, 29]

(12) $$\begin{array}{c} S_{u}\left(n\right)=\frac{2\bar{u^{\prime 2}}L_{u}^{x}}{\bar{U}{\left[1+{\left(2cnL_{u}^{x}/\bar{U}\right)}^{2}\right]}^{5/6}}, \end{array}$$

where $\bar{U}$ is mean wind speed; *L*_*x*_^*u*^ is longitudinal turbulence integral scale; $\bar{u^{\prime 2}}$ is standard deviation of the longitudinal fluctuating component; and constant coefficient *c* is 4.2065.

Power spectral densities of the longitudinal and lateral fluctuating components are derived from the same characteristic of turbulence hypothesis:

(13) $$\begin{array}{c} S_{v}\left(n\right)=S_{w}\left(n\right)=\frac{1}{2}\left[S_{u}\left(n\right)-\frac{ndS_{u}\left(n\right)}{dn}\right]. \end{array}$$

For isotropic turbulences, $\bar{v^{\prime 2}}=\bar{w^{\prime 2}}=\bar{u^{\prime 2}}$, *L*_*u*_^*x*^ = *L*_*v*_^*y*^ = *L*_*w*_^*z*^, some equations are obtained from plugging (12) into (13):

(14) $$\begin{array}{c} S_{v}\left(n\right)=\frac{\bar{v^{\prime 2}}L_{v}^{y}\left[1+\left(8/3\right){\left(cnL_{v}^{y}/\bar{U}\right)}^{2}\right]}{\bar{U}{\left[1+{\left(2cnL_{v}^{y}/\bar{U}\right)}^{2}\right]}^{11/6}},\\ S_{w}\left(n\right)=\frac{\bar{w^{\prime 2}}L_{w}^{z}\left[1+\left(8/3\right){\left(cnL_{w}^{z}/\bar{U}\right)}^{2}\right]}{\bar{U}{\left[1+{\left(2cnL_{w}^{z}/\bar{U}\right)}^{2}\right]}^{11/6}}. \end{array}$$

If *L*_*v*_^*x*^ = *L*_*w*_^*x*^ = 0.5*L*_*u*_^*x*^ for isotropic turbulences, the expressions of Von Karman spectrum are derived from plugging *L*_*v*_^*x*^ = *L*_*w*_^*x*^ = 0.5*L*_*u*_^*x*^ into (14):

(15) nSu(n)σu2=4f(1+70.8f2)5/6,nSi(n)σi2=4f(1+755.2f2)(1+283.2f2)11/6, i=v,w.

**Figure 18:**
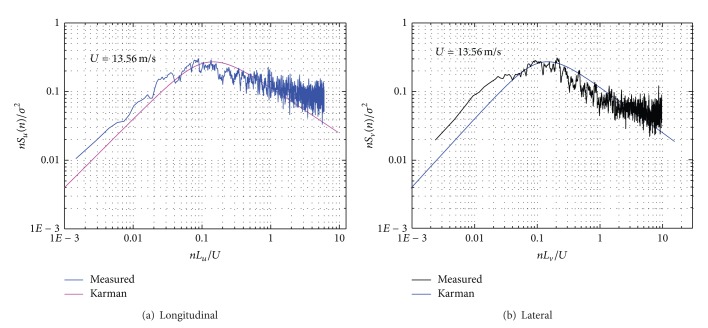
Power spectral density of fluctuation wind speed components (*U* = 13.56 m/s).

**Figure 19:**
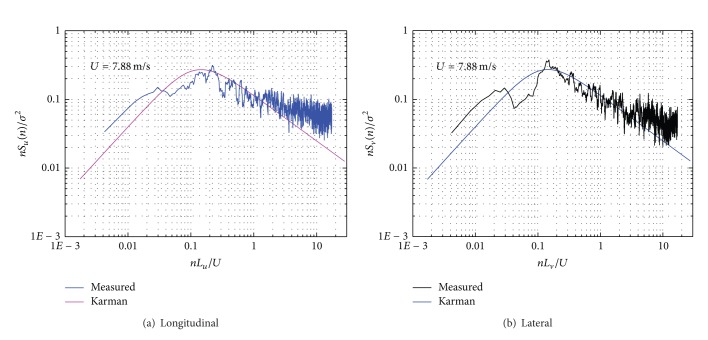
Power spectral density of fluctuation wind speed components (*U* = 7.88 m/s).

In Figures 18 and 19, field-measured power spectral densities of longitudinal and lateral fluctuate components are shown at wind speed of 13.56 m/s and 7.88 m/s, presenting a good agreement with the Karman empirical spectra for the whole set of data. However, the PSD data estimated by field measurement are slightly larger than that by empirical method when the reduced frequency *nz*/*U*(*z*) is smaller than 0.1 or larger than 0.1.

## 4. Conclusions

Through a full-scale wind speed and direction monitoring system on Jiubao Bridge in Hangzhou city, this paper presents a reliable study of wind speed and direction characteristics during typhoon HAIKUI, such as time histories of mean wind speed and direction, turbulence intensity, peak factor, gust factor, probability distribution of fluctuating velocity component, correlation among fluctuating velocities, turbulence integral scale, and the power spectrum of fluctuating velocity, and the results are summarized as follows.

1. Turbulence intensities decrease with increased anemometer elevation for both longitudinal and lateral components of mean wind speed. A remarkable decrease of turbulence intensity is shown with increased mean wind speed, typically at low mean wind speeds (*u* < 8 m/s), but an unknown tendency when mean wind speed exceeds 8 m/s. The ratio of turbulence intensities of longitudinal component to lateral component is 0.85 in this paper, indicating a clear similarity with the results by Tieleman, Cao, and Bao-Shi, whereas a certain deviation from Huang et al.'s results [11].
2. Gust factors decrease with mean wind speed. An obvious decrease of the change rate is found at low wind speeds, but wind speed seems to have less impact on the change rate when it becomes high. The average ratio for gust factor of longitudinal component to lateral component is 0.30. The upper and lower peak factors are recorded as 0.6 and 2.5; the average and standard deviation are 1.52 and 0.34, respectively. A noticeable decrease is found from the analysis of the average peak factor compared with Peng et al.'s results [12].
3. Probability density distribution of wind speed fluctuations at different wind speeds agrees well with a Gaussian distribution, which means the full-measured recorded data of wind speed fluctuations are in accordance with Gauss assumption.
4. Increasing quantity of turbulence integral scales is recorded by increased 10 min mean wind speed. A similarity of probability distributions of turbulence integral scales of the three components is that each distribution is obviously asymmetrical and does not follow a standardized Gaussian distribution.
5. Power spectral densities of recorded wind speed fluctuations are fairly good in agreement with Von Karman spectrum.

## Figures and Tables

**Table 1:** Specifications of anemometer.

Brand (type)	Wind speed		Wind direction		Measurement and structure	
Gill Windsonic	Range	0–60 m/s (116 Knots)	Range	0–359°	Frequency	0.25 Hz, 0.5 Hz, 1 Hz, 2 Hz or 4 Hz
	Accuracy	±2%; 12 m/s	Accuracy	±3° at 12 m/s	Parameter	U and V
	Resolution	0.01 m/s (0.02 Knots)	Resolution	1°	Measurement unit	m/s, knots, mph, kph, ft/min
	Reaction time	0.25 s	Reaction time	0.25 s	Size	142 mm × 160 mm
	Lowest	0.01 m/s	/	/	Wight	0.5 Kg

**Table 2:** Ratios of turbulence intensity among the turbulence components.

Research man	Wind	Height (m)	*I*_*u*_ : *I*_*v*_	Sites
Tieleman [27]	Normal strong wind	5	1 : 0.80	Holland

Cao et al. [6]	Typhoon Maemi	10	1 : 0.83	Japan

Shiau	Typhoon Zeb [24]	26	1 : 0.98	Taiwan, China
	Typhoon Babs [25]		1 : 0.78	
	Normal strong wind [26]		1 : 0.78	

Fu et al. (2008) [23]	Typhoon Sanvu	10	1 : 0.7	Guangzhou, China

Peng et al. [12]	Typhoon Muifa	10	1 : 0.66	Shanghai, China
		20	1 : 0.65	
		40	1 : 0.74	

Present test	Typhoon HAIKUI	6 (upper bridge)	1 : 0.85	Hangzhou, China
